# Metagenomic Insight into the Effect of Probiotics on Nitrogen Cycle in the *Coilia nasus* Aquaculture Pond Water

**DOI:** 10.3390/microorganisms12030627

**Published:** 2024-03-21

**Authors:** Qi Mang, Jun Gao, Quanjie Li, Yi Sun, Gangchun Xu, Pao Xu

**Affiliations:** 1 Wuxi Fisheries College, Nanjing Agricultural University, Wuxi 214081, China; mangq@cafs.ac.cn; 2 Key Laboratory of Freshwater Fisheries and Germplasm Resources Utilization, Ministry of Agriculture, Freshwater Fisheries Research Center, Chinese Academy of Fishery Sciences, Wuxi 214081, China; gaojun@ffrc.cn (J.G.); liqj@ffrc.cn (Q.L.); sunyi@ffrc.cn (Y.S.)

**Keywords:** probiotics, ammonia, nitrite, nitrogen cycling, nitrification, denitrification

## Abstract

Recently, probiotics have been widely applied for the in situ remediation of aquatic water. Numerous studies have proved that probiotics can regulate water quality by improving the microbial community. Nitrogen cycling, induced by microorganisms, is a crucial process for maintaining the balance of the aquatic ecosystem. Nevertheless, the underlying mechanisms by which probiotics enhance water quality in aquatic systems remain poorly understood. To explore the water quality indicators and their correlation with nitrogen cycling-related functional genes, metagenomic analysis of element cycling was performed to identify nitrogen cycling-related functional genes in *Coilia nasus* aquatic water between the control group (C) and the groups supplemented with probiotics in feed (PF) or water (PW). The results showed that adding probiotics to the aquatic water could reduce the concentrations of ammonia nitrogen (NH_4_^+^-N), nitrite (NO_2_^−^-N), and total nitrogen (TN) in the water. Community structure analysis revealed that the relative abundance of Verrucomicrobiota was increased from 30 d to 120 d (2.61% to 6.35%) in the PW group, while the relative abundance of Cyanobacteria was decreased from 30 d to 120 d (5.66% to 1.77%). We constructed a nitrogen cycling pathway diagram for *C. nasus* aquaculture ponds. The nitrogen cycle functional analysis showed that adding probiotics to the water could increase the relative abundance of the *amoC_B* and *hao* (Nitrification pathways) and the *nirS* and *nosZ* (Denitrification pathways). Correlation analysis revealed that NH_4_^+^-N was significantly negatively correlated with *Limnohabitans*, *Sediminibacterium*, and *Algoriphagus*, while NO_2_^−^-N was significantly negatively correlated with *Roseomonas* and *Rubrivivax*. Our study demonstrated that adding probiotics to the water can promote nitrogen element conversion and migration, facilitate nitrogen cycling, benefit ecological environment protection, and remove nitrogen-containing compounds in aquaculture systems by altering the relative abundance of nitrogen cycling-related functional genes and microorganisms.

## 1. Introduction

Aquatic animals are an important food source for humans. Pond aquaculture is a primary method of freshwater aquaculture [1]. However, with the continuous expansion of aquaculture, the environmental issues have worsened gradually [2]. The decomposition of a large amount of feces and uneaten feed leads to an increase in the concentration of various forms of nitrogen in the water, especially ammonia nitrogen (NH_4_^+^-N) and nitrite (NO_2_^−^-N) [3]. NH_4_^+^-N and NO_2_^−^-N present substantial hazards to aquatic animals, directly impacting their health and growth [4,5,6]. Additionally, the elevation of total nitrogen content in the aquaculture water can disturb the nitrogen-phosphorus ratio, consequently leading to pond eutrophication [7].

Microbial communities play a crucial role in the elemental cycling and energy flow processes within aquatic ecosystems [8,9]. Nitrifying bacteria and denitrifying bacteria, among other microorganisms, are essential for removing nitrogen from the water. Nitrogen cycling is one of the most important biogeochemical cycles in the Earth’s ecosystem and has received widespread attention in ecological and environmental research [10,11]. Over the past few decades, nitrogen cycling pathways have been extensively studied in different ecosystems. In recent years, metagenomic sequencing has been applied to explore nitrogen cycle-related gene families and link them to environmental factors [12,13,14,15]. However, most current research on microbial communities in aquaculture water focuses solely on simple correlation analysis between microorganisms and water quality indicators, neglecting the role of microorganisms in element cycling processes, particularly nitrogen cycling.

Probiotics are widely used in agriculture, animal husbandry, aquaculture, environmental management, and other fields [16,17,18]. They can help maintain microbial balance, promote the proliferation of beneficial microorganisms, and suppress the growth of pathogenic microorganisms, thereby improving water quality and promoting the healthy growth of aquatic animals [19]. Additionally, probiotics contribute to the decomposition of organic waste and facilitate the conversion of elements, especially nitrogen, during the cycling process, thereby supporting the preservation of ecological equilibrium in aquatic environments [20,21]. In the field of aquaculture, probiotics are frequently administered through two methods: incorporation into feed or addition to the water system. Both approaches have been shown to yield positive outcomes in terms of improving the water quality and overall performance of the aquaculture system. It has been reported that adding probiotics can mitigate nitrogenous compounds (NH_4_^+^-N, NO_2_^−^-N, and NO_3_^−^-N) and phosphorus compounds, as well as modulate the microbial community structure [22,23].

*Coilia nasus*, a highly prized and delectable species, is extensively cultivated in East Asia. Nevertheless, intensive feeding during *C. nasus* production has led to the accumulation of excessive ammonia nitrogen, posing a significant risk to survival and growth [24]. The utilization of probiotic supplementation in aquaculture is progressively gaining prevalence. Nevertheless, most of the current research on microbial communities in aquaculture water systems focuses solely on simple correlation analyses between microorganisms and water quality indicators, overlooking the important role of microorganisms in element cycling processes, particularly nitrogen cycling.

To investigate the impact of probiotics on microbial diversity and community composition associated with nitrogen cycling in *C. nasus* aquaculture ponds, we seek to unravel the potential mechanisms underlying the nitrogen removal efficacy of probiotics via metagenomic analysis of element cycling. This investigation will contribute novel perspectives into the nitrogen removal capabilities of probiotics and provide essential insights for mitigating nutrient loading in fish farming ponds.

## 2. Materials and Methods

### 2.1. Experimental Design, Sampling, and Water Quality Determination

Healthy *C. nasus* individuals (11.34 ± 1.16 cm, 5.82 ± 0.84 g) were sourced from Yangzhong, China. A total of nine ponds (160 m^3^) were divided into three groups (300 individuals per pond): the control group (C), adding probiotics in feed group (PF), and adding probiotics in water group (PW). The fish underwent a 7-day acclimation period before the commencement of the experiment. The C group received no probiotics in a basal diet or aquatic water. The PF group was given 1.0 × 10^8^ CFU/g of effective microorganisms (EM) in a basal diet, based on other references and our previous study [25,26]. The PW group was also provided with 1.0 × 10^8^ CFU/g of EM (every 4 days), following other references and our previous study [27]. The experimental period lasted for 120 days (from April to August), and continuous microbubble aeration was employed throughout the aquaculture period. Sampling was conducted once every 30 days. Water samples were collected via a five-point sampling method from 50 cm below the surface of the water. Each water sample was mixed thoroughly, and 1.0 L was used to measure water quality indicators, whereas 3.0 L was promptly filtered through 0.22 μm polycarbonate membranes for metagenomic analysis. The detailed contents of effective microorganisms (Hengtai Biotechnology Co., Ltd., Wuxi, China) and dairy diets (Tianen Aquatic Feed Co., Ltd., Jiaxing, China) used are shown in Tables S1 and S2. The water quality indicators were analyzed following the methods described by [28]. The concentration of NH_4_^+^-N in the water was determined via the Nessler’s reagent spectrophotometric method. NO_2_^−^-N was quantified using the spectrophotometric method, while nitrate nitrogen (NO_3_^−^-N) was analyzed via the zinc-cadmium reduction method. Total nitrogen (TN) levels were measured using the alkaline potassium persulfate digestion method in combination with ultraviolet spectrophotometry.

### 2.2. DNA Extraction and Metagenomic Sequencing

Microbial DNA was isolated from water samples using the E.Z.N.A.^®^ stool DNA Kit (Omega Bio-tek, Norcross, GA, USA) following the manufacturer’s instructions. Subsequently, metagenomic shotgun sequencing libraries were prepared and sequenced at Shanghai Biozeron Biological Technology Co., Ltd., Shanghai, China. Briefly, genomic DNA (1 μg) was fragmented using a Covaris S220 Focused-ultrasonicator (Woburn, MA, USA), and sequencing libraries with an average fragment length of approximately 450 bp were generated. All samples were sequenced on the Illumina NovaSeq 6000 platform in paired-end 150 bp (PE150) mode. Raw sequence reads underwent quality trimming using Trimmomatic v0.36 (http://www.usadellab.org/cms/uploads/supplementary/Trimmomatic, accessed on 16 June 2023) to remove adapter contaminants and low-quality reads.

### 2.3. Reads-Based Phylogenetic Annotation

The taxonomy of clean reads for each sample was determined using Kraken2 with a customized kraken database. This customized database consisted of genome sequences from bacteria, archaea, fungi, viruses, protozoa, and algae obtained from the NCBI RefSeq database (release number: 20221209). The classification of reads was performed at seven phylogenetic levels, including domain, phylum, class, order, family, genus, and species, as well as an “unclassified” category. To estimate the abundances of different taxa, Bracken v2.7.0 (https://ccb.jhu.edu/software/bracken/, accessed on 16 June 2023) was employed. Bracken is capable of providing accurate estimations of species- and genus-level abundance, even in cases where there are multiple closely related species. The relative abundance of a certain taxonomic level in the study represents the cumulative abundance of species belonging to that specific level.

### 2.4. Metagenomic De Novo Assembly, Gene Prediction, Gene Abundance

The clean sequence reads were used to generate a set of contigs for each sample by employing MegaHit (v1.1.1-2-g02102e1) with the parameter “--min-contig-len 500”. Subsequently, the open reading frames (ORFs) within the assembled contigs were predicted using METAProdigal (v2.6.3). All ORFs were then clustered using CD-HIT to generate a set of unique genes. The longest sequence within each cluster was chosen as the representative sequence for each gene in the unique-gene set. To determine the abundance profiles of these genes, the high-quality reads from each sample were aligned against the unique-gene set using BWA-MEM (v.0.7.17). Abundance values for the genes were calculated in transcripts per million (TPM), taking into account variations in gene length and the number of mapped reads per sample. Specifically, genes with an alignment length of at least 50 bp and a sequence identity higher than 95% were included in the abundance calculations.

### 2.5. Gene Function Annotation Based on Unique Gene

The unique-gene set was searched against various databases to identify proteins and retrieve their functional annotations. The KEGG database was used with kofam v1.2.0, while the Carbohydrate-Active Enzymes (CAZy v8) database and eggNOG v5.0 database were compared using BLASTP searches against the NCBI NR database with DIAMOND (v0.9.22.123). To identify antibiotic resistance genes (ARGs), the SARG v2.3 database was used with diamond (v0.9.22.123) BLASP (http://blast.ncbi.nlm.nih.gov/Blast.cgi, accessed on 17 June 2023), requiring an identity ≥ 80% and coverage ≥ 70% for identifying ARG-like ORFs. Pathogens and virulence factor (VF) gene annotations were conducted by aligning amino acid sequences with the PHI database (http://www.phi-base.org/index.jsp, accessed on 17 June 2023) and VFDB database (http://www.mgc.ac.cn/VFs/, accessed on 17 June 2023) using BLASTP with an e-value cutoff of 10^−5^ and identity ≥ 70%. For the BactMet gene annotations, amino acid sequences were aligned with the BacMet V2 database (http://bacmet.biomedicine.gu.se/, accessed on 17 June 2023) using BLASTP with an e-value cutoff of 10^−5^ and identity ≥ 70%. We utilized a high-quality reference database, the NCyc database (https://github.com/qichao1984/NCyc/tree/master/data, accessed on 17 June 2023), for the metagenomic analysis of nitrogen cycling gene families [29].

### 2.6. Statistical Analysis

To assess the normal distribution of the data, the Kolmogorov-Smirnov and Shapiro-Wilk methods were employed. In cases where the data deviated from normal distribution, a conversely non-normally distributed data approach was utilized to test for interactive effects. If *p* < 0.05, a two-way ANOVA was conducted using SPSS 20.0 software. The mean values, accompanied by the standard error of the mean (SEM), were presented for all data. A *p* < 0.05 was considered statistically significant. Line charts were drawn via GraphPad 10.0. The heatmaps were drawn via TBtools [30]. The PCoA, correlation heatmaps, and RDA analysis were performed on Omicshare Tools (https://www.omicshare.com/tools/, accessed on 10 December 2023) [31,32].

## 3. Results

### 3.1. Characteristics of Nitrogen Element Transformation in Different Forms

**Figure 1:**
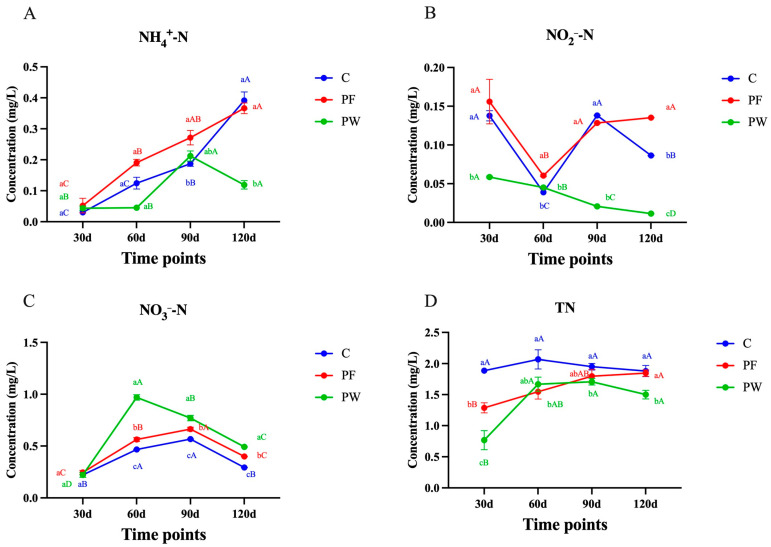
Chemical characteristics of nitrogen elements in a pond. (**A**) ammonia nitrogen (NH_4_^+^-N), (**B**) nitrite (NO_2_^−^-N), (**C**) nitrate (NO_3_^−^-N), and (**D**) total nitrogen (TN). Different capital letters indicate significant differences among different time points in the same groups (*p* < 0.05). Different lower-case letters indicate a significant difference between different groups at the same time point (*p* < 0.05).

The concentration of NH_4_^+^-N in both the C group and PF group showed an increasing trend. However, there was no significant difference between these two groups at 120 days (*p* > 0.05). On the other hand, the concentration of NH_4_^+^-N in the PW group was significantly lower than that in the C group and PF group at both 60 and 120 days (*p* < 0.05) (Figure 1A). At 120 days, the PF group demonstrated significantly lower NO_2_^−^-N levels than the C group (*p* < 0.05). Furthermore, both the C and PF groups had significantly higher NO_2_^−^-N levels compared to the PW group at 30, 90, and 120 days (*p* < 0.05) (Figure 1B). The NO_3_^−^-N levels in the C, PF, and PW groups displayed similar trends, initially increasing and then decreasing. At 60, 90, and 120 days, the PW group exhibited the highest NO_3_^−^-N content, surpassing the other two groups with statistical significance (*p* < 0.05). Conversely, the PF group consistently had the lowest NO_3_^−^-N content at each time point, significantly lower than the other two groups (*p* < 0.05) (Figure 1C). Regarding TN content, the PW group consistently had significantly lower levels compared to the C group at all time points (*p* < 0.05). Additionally, the PF group had significantly lower TN content than the C group at 30 and 60 days (*p* < 0.05) (Figure 1D).

### 3.2. Characteristics of Microbial Diversity and Community Structure Differences

**Figure 2:**
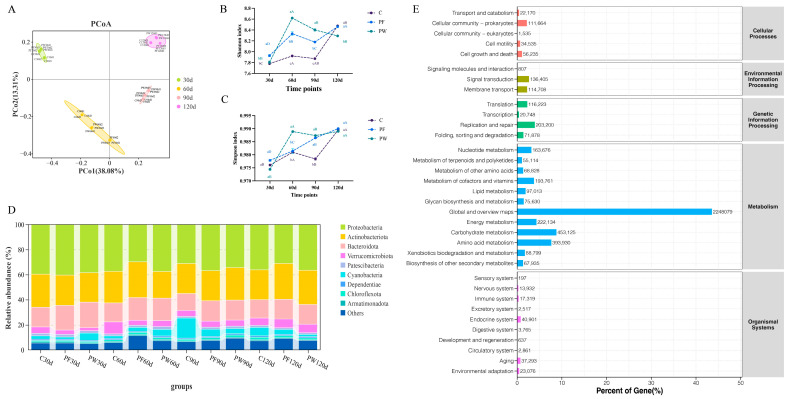
Differences in microbial diversity and community structure. PCoA analysis (**A**), shannon index (**B**), simpson index (**C**), characteristics of bacterial distribution at phylum level (**D**), and KEGG functional enrichment (**E**). Different capital letters indicate significant differences among different time points in the same groups (*p* < 0.05). Different lower-case letters indicate a significant difference between different groups at the same time point (*p* < 0.05).

After filtering adaptor sequences, ambiguous ‘N’ nucleotides, and low-quality sequences, a total of 786,632,238, 788,392,474, and 713,383,154 clean reads were generated in the C, PF, and PW groups, respectively (Table S3). After metagenome assembly, a total of 4,830,075, 5,572,128, and 5,102,814 contigs sequences were obtained in the C, PF, and PW groups, respectively (Table S4). The principal coordinate analysis (PCoA) demonstrated significant differences in microbial community structure across various time points (Figure 2A).

In comparison to the C group, both the PF group and PW group exhibited a significantly upward trend in microbial α-diversity (Shannon index and Simpson index) (*p* < 0.05) (Figure 2B,C). Proteobacteria (29.76% to 40.39%), Actinobacteria (21.24% to 28.64%), and Bacteroidetes (13.52% to 20.21%) were the predominant bacterial phyla observed in the aquaculture water of *C. nasus* at the phylum level, accounting for more than 60% of the relative abundance and displaying absolute predominance (Figure 2D). The relative abundance of Cyanobacteria increased over time in the C and PF groups, but decreased in the PW group from 30 d to 120 d (5.66% to 1.77%). On the other hand, the relative abundance of Verrucomicrobiota increased from 30 d to 120 d (2.61% to 6.35%) (Figure 2D). Moreover, the relative abundance of the metabolism pathway was dominant based on KEGG (Figure 2E).

### 3.3. Nitrogen Cycling Pathways and Their Key Functional Genes

**Figure 3:**
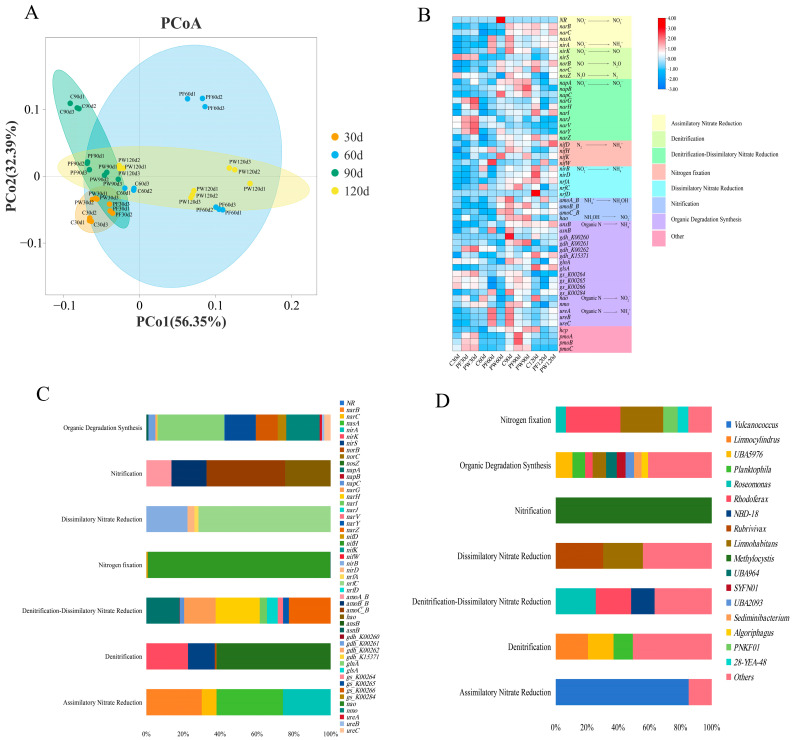
Analysis of genes associated with nitrogen cycling pathways. PCoA analysis (**A**), relative abundance of genes associated with nitrogen cycling pathways (**B**), contribution of genes related to nitrogen cycling pathways (**C**), contribution of genus related to nitrogen cycling pathways (**D**).

The PCoA analysis indicated no significant differences in nitrogen cycling-related genes among different time points (Figure 3A). As shown in Figure 3B, a total of 54 genes involved in nitrogen cycling were identified based on the Ncycle database. These genes participate in the following nitrogen cycling pathways: Assimilatory Nitrate Reduction (ANR), Denitrification, Denitrification-Dissimilatory Nitrate Reduction (DDNR), Nitrogen fixation (NF), Dissimilatory Nitrate Reduction (DNR), Nitrification, and Organic Degradation Synthesis (ODS). The contribution of genes and microbial taxa (genus level) involved in each nitrogen cycling pathway is depicted in Figure 3C (Table S5) and Figure 3D (Table S6). *nirA*, *nasA*, *narB*, and *Vulcanococcus* exhibited the highest contribution in the ANR. *nosZ*, *nirK*, *nirS*, and *Limnocylindrus* demonstrated the highest contribution to Denitrification. *napA*, *narG*, *narZ*, and *Roseomonas*, *Rhodofera* showed the highest contribution in DDNR. *nifH* and *Rhodoferax*, *Limnohabitans* exhibited the highest contribution in NF. *nirB*, *nrfC*, and *Rubrivivax*, *Limnohabitans* demonstrated the highest contribution in DNR. *amoC_B*, *hao*, and *Methylocystis* showed the highest contribution to Nitrification. *nmo*, *glnA*, *gs_K00265*, *gs_K00266*, and *UBA5976*, *Limnohabitans*, *Planktophila* exhibited the highest contribution in ODS. However, in this study, functional genes responsible for the oxidation of nitrite to nitrate were not identified in the nitrogen cycling pathway of the *C. nasus* aquaculture water.

**Figure 4:**
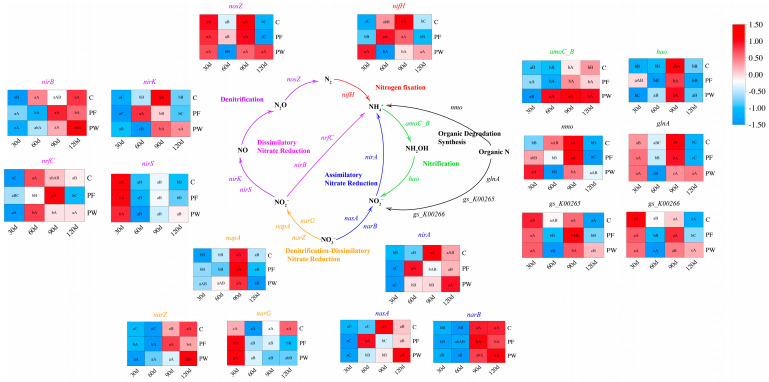
Construction of nitrogen cycling pathways and relative abundance of genes involved in nitrogen cycling pathways. Different capital letters indicate significant differences among different time points in the same groups (*p* < 0.05). Different lower-case letters indicate a significant difference between different groups at the same time point (*p* < 0.05).

Based on the function of nitrogen cycling genes, we constructed a nitrogen cycling pathway (Figure 4). Compared to the C group, the PF group and the PW group had a significant impact on the relative abundance of nitrogen cycling genes. After 30 days, the relative abundance of DDNR (*napA*, *narG*, *narZ*) and ANR (*nirA*, *narB*) in the PW group was significantly increased (*p* < 0.05). After 60 days, the relative abundance of DNR (*nirB*, *nrfC*) and ODS (*nmo*, *glnA*, *gs_K00265*, *gs_K00266*) in the PW group was significantly decreased (*p* < 0.05), while the relative abundance of DDNR (*napA*, *narZ*), ANR (*nirA*, *narB*, *nasA*), and Nitrification (*amoC_B*, *hao*) in the PW group was significantly up-regulated (*p* < 0.05). After 90 days, the relative abundance of ANR (*nirA*, *nasA*), and ODS (*nmo*, *glnA*, *gs_K00265*)-related genes in the PW group was significantly down-regulated (*p* < 0.05). However, the relative abundance of Nitrification (*amoC_B*, *hao*) in the PW group was significantly enhanced (*p* < 0.05). After 120 days, the relative abundance of Denitrification (*nosZ*, *nirK*, *nirS*), and ODS (*nmo*, *glnA*)-related genes in the PW group was significantly promoted (*p* < 0.05).

### 3.4. Correlation Analysis of Key Functional Genes, Microorganisms, and Water Quality in Nitrogen Cycling Pathways

**Figure 5:**
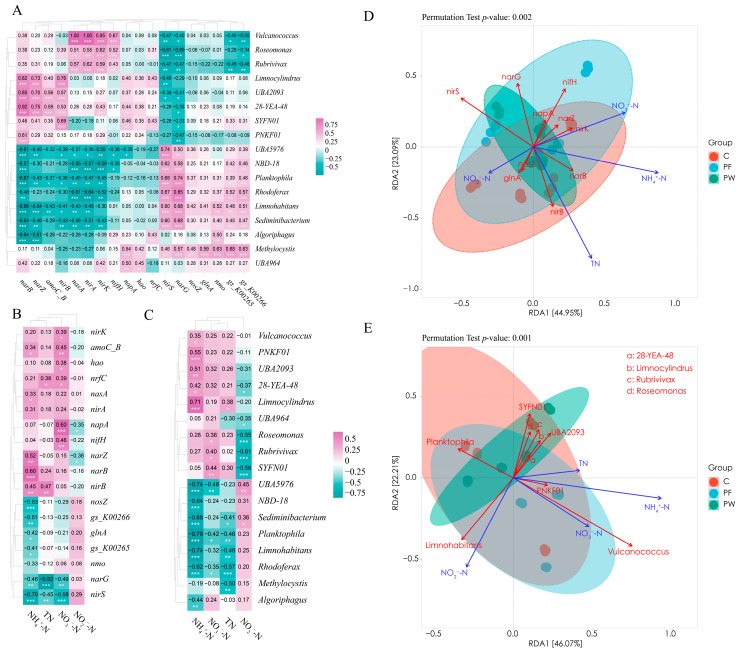
Correlation analysis of genes, genus, and water quality related to nitrogen cycling pathways. Correlation heatmap of genes and genus related to nitrogen cycling pathways (**A**), correlation heatmap of genes and water quality related to nitrogen cycling pathways (**B**), correlation heatmap of genus and water quality related to nitrogen cycling pathways (**C**), RDA analysis of genes and water quality related to nitrogen cycling pathways (**D**), and RDA analysis of genus and water quality related to nitrogen cycling pathways (**E**). The difference was considered statistically significant when * *p* < 0.05, ** *p* < 0.01, or *** *p* < 0.001.

The correlation heatmap analysis showed a correlation between “microbiome-functional genes-water quality indicators”. Figure 5A presents the relationship between functional genes and the microbiota. The results indicated a consistent correlation between the functional genes involved in nitrogen cycling and the microbiota. Specifically, the functional genes related to ANR (*nirA*, *nasA*, *narB*), Denitrification (*nosZ*, *nirK*, *nirS*), and ODS (*gs_K00265*, *gs_K00266*) showed similar associations.

In Figure 5B, the relationship between functional genes and water quality indicators was examined. The findings revealed a significant negative correlation between ammonia nitrogen and Denitrification (*nirS*, *nosZ*), DDNR (*narG*), and ODS (*glnA*, *gs_K00265*, *gs_K00266*). Additionally, there was a significant negative correlation observed between nitrite and the DDNR (*napA*, *narZ*). Moreover, a significant positive correlation was identified between nitrate and Denitrification (*nirK*), DDNR (*napA*), and DNR (*nrfC*). 

In Figure 5C, the relationship between microbiota and water quality indicators was examined. The findings revealed a significant negative correlation between ammonia nitrogen and the relative abundance of *Rhodoferax*, *Limnohabitans*, *Sediminibacterium*, and *Algoriphagus*. Additionally, there was a significant negative correlation observed between nitrite and the relative abundance of *Roseomonas* and *Rubrivivax*. Moreover, a significant negative correlation was identified between nitrate and the relative abundance of *Planktophila* and *Rhodoferax*. 

Moreover, RDA analysis was employed to examine the environmental factors that impact the regulation of nitrogen cycling-related functional genes and microbiota by probiotics. The length of the arrows in Figure 5D represents the significance of TN, NH_4_^+^-N, and NO_3_^−^-N as influential environmental factors in explaining the regulation of nitrogen cycling-related functional genes by probiotics. Similarly, the length of the arrows in Figure 5E indicates the importance of NH_4_^+^-N, NO_2_^−^-N, and NO_3_^−^-N in elucidating the regulation of nitrogen cycling-related microbiota by probiotics.

## 4. Discussion

The rapid expansion and intensification of aquaculture have resulted in excrement and residual feed becoming significant contributors to excessive organic and inorganic nutrients in aquaculture water. This disturbance in the equilibrium of aquatic ecosystems has led to eutrophication, posing a threat to the health of aquatic animals [33,34]. Elevated concentrations of NH_4_^+^-N, NO_2_^−^-N, and NO_3_^−^-N can have detrimental effects on the respiratory, nervous, immune, and growth functions of aquatic organisms [35,36,37,38]. In addition, excessive TN content in water can trigger eutrophication. Therefore, maintaining a nitrogen balance in aquaculture water is an important way to achieve green aquaculture and ensure the welfare of aquatic animals. Increasing research has shown that probiotics applied for regulation can effectively remove nutrients from water, especially nitrogen, and inhibit eutrophication [39,40,41]. In crucian carp (*Carassius auratus gibelio*), exposure to high concentrations of complex probiotics resulted in the attainment of a dynamic equilibrium state for total ammonia nitrogen, and nitrite levels [42]. The mixed probiotics have shown positive effects on reducing nitrogen and phosphorus compounds [22]. Microencapsulated Bacillus probiotic significantly reduced the levels of NH_4_^+^-N and NO_2_^−^-N in white shrimp (*Litopenaeus vannamei*) [43]. Consistent with previous studies, the present study demonstrated that adding probiotics to water significantly reduced the levels of NH_4_^+^-N, NO_2_^−^-N, and TN content. Adding *Bacillus subtilis SC02* to *Ctenopharyngodon idellus* culture improved water quality, possibly due to changes in microbial community diversity [44]. Furthermore, this study demonstrated that adding probiotics to water altered the structure of microbial communities, reducing the relative abundance of Cyanobacteria and increasing the relative abundance of Verrucomicrobiota. Cyanobacteria exhibit a preference for inorganic forms of nitrogen, particularly NH_4_^+^-N and NO_2_^−^-N [45]. It has been found that Verrucomicrobiota can inhibit the growth of cyanobacteria by competing for nutrients and producing inhibitory metabolites [46,47]. These results suggest that adding probiotics to water may inhibit Cyanobacteria by increasing the relative abundance of Verrucomicrobiota and reducing the concentrations of NH_4_^+^-N and NO_2_^−^-N in the water. 

The nitrogen cycle is critical to preserving nitrogen balance and maintaining ecosystem stability in water by regulating nitrogen levels and preventing eutrophication [48]. Microorganisms serve as important drivers of nitrogen cycling processes in aquatic environments. The present study constructed the nitrogen cycling pathways in *C. nasus* aquaculture water based on metagenomic analysis, including Assimilatory Nitrate Reduction, Denitrification, Denitrification-Dissimilatory Nitrate Reduction, Nitrogen fixation, Dissimilatory Nitrate Reduction, Nitrification, and Organic Degradation Synthesis, which was consistent with previous studies [49]. Our previous studies have indicated that elevated levels of NH_4_^+^-N can be detrimental to the health of *C. nasus*, leading to inflammatory responses, immune suppression, and neurological damage [24]. In the present study, adding probiotics in water significantly reduced the concentration of NH_4_^+^-N in *C. nasus* aquaculture water. Nitrification is a vital mechanism for the elimination of NH_4_^+^-N, with ammonia oxidation serving as a crucial rate-limiting step in this process. *AmoC_B* is an important subunit of ammonia monooxygenase, which participates in the oxidation of ammonia nitrogen to NH_2_OH [50,51]. The nitrite oxidoreductase encoded by the *hao* gene is involved in the oxidation of NH_2_OH to nitrite in the nitrogen cycle and serves as a key regulatory factor in the process [52]. In the present study, the relative abundance of *amoC_B* and *hao* genes was higher in the group with adding probiotics compared to other groups. This indicates that adding probiotics to water enhances the potential for Nitrification. In addition, this study found a significant negative correlation between the concentration of NH_4_^+^-N and the relative abundance of *Limnohabitans*, *Sediminibacterium*, and *Algoriphagus* in the water bodies. Some species within the *Limnohabitans* possess ammonia monooxygenase genes and have been shown to have the ability to utilize ammonia nitrogen and convert it to nitrite through the process of ammonification [53]. *Sediminibacterium* is responsible for denitrification in sludge [54]. An increased nutrient concentration significantly affected bacterial abundance. *Sediminibacterium* showed an evident response to high nutrient concentrations [55]. These results suggest that adding probiotics to water may regulate the concentration of ammonia nitrogen by altering the relative abundance of water body microorganisms and Nitrification-related genes.

ANR, Denitrification, DDNR, and DNR are important pathways for removing NO_2_^−^-N, and NO_3_^−^-N from water. They also play a crucial role in biological denitrification in wastewater treatment processes [56]. In the present study, the relative abundance of *nirS* and *nosZ* genes involved in Denitrification was higher in the group with probiotics compared to other groups. However, the relative abundance of *nirB*, *nrfC* (DNR), and *nirA* (ANR) was lower in the group with probiotic addition compared to other groups. The *nirS* and *nosZ* genes encode nitrite reductase enzymes that catalyze the reduction in nitrite to nitrogen gas using an electron donor. This process is an important step in the nitrogen cycle and helps maintain the balance of nitrogen elements in ecosystems [57,58]. In addition, in this study, functional genes responsible for the oxidation of nitrite to nitrate were not identified in *C. nasus* aquaculture water. These results suggest that adding probiotics to water primarily removes NO_2_^−^-N from water through the Denitrification pathway. In this study, correlation analysis indicated a significant negative correlation between nitrite and the genera *Roseomonas* and *Rubrivivax*. Adding *Bacillus subtilis SC02* to *Ctenopharyngodon idellus* culture can improve water quality and increase the relative abundance of *Roseomonas* [44]. In a study on treating coking wastewater, *Rubrivivax* was proven to be responsible for denitrification [59]. These reports are similar to the results of this study. These findings suggest that adding probiotics to water may regulate nitrite levels in water by altering the relative abundance of water microbial communities and denitrification-related genes. Additionally, in this study, the relative abundance of *napA*, *narZ*, and *narG* genes associated with Dissimilatory Nitrate Reduction to Ammonium (DDNR) was higher in the group with probiotics compared to other groups. This increase in gene abundance is correlated with the nitrate concentration in the water. The key genes *napA*, *narZ*, and *narG* are subunits of nitrate assimilation reductases involved in the process of DDNR. They play an important role in the nitrogen cycle [60,61]. The results indicate that an increase in nitrate concentration in water can promote the abundance of nitrate-related nitrogen cycling genes. This is likely due to the fact that the rise in nitrate levels may stimulate the growth and reproduction of nitrifying bacteria, resulting in an increase in the abundance of nitrification-related genes. Moreover, the increase in nitrate concentration may also trigger the metabolic activity of denitrifying bacteria, leading to an increase in the abundance of denitrification-related genes, which is consistent with the findings of this study. In aquaculture practices, a large amount of nutrients is typically stored in sediments [62]. In the study of zero-water exchange ponds, nitrate concentrations in both water and sediments significantly increased over time [63]. The nitrate in sedimentary deposits is not permanently fixed within them; under certain conditions, it can be released back into the water. Therefore, we speculate that the reason for the higher nitrate levels in the water after adding probiotics in this study may be related to sediment release. However, the specific mechanisms still need to be verified and further investigated.

## 5. Conclusions

The results of this study indicate that, compared to adding probiotics in feed, adding probiotics in water significantly reduced the concentrations of ammonia nitrogen, nitrite, and total nitrogen in the water. Through metagenomic sequencing of microorganisms in the water and nitrogen cycle analysis, we constructed a nitrogen cycle pathway diagram for *C. nasus* aquaculture water. Adding probiotics to water increases the relative abundance of the *amoC_B* and *hao* (Nitrification pathways) at 60 d and 120 d, as well as *nirS* and *nosZ* (Denitrification pathways) at 120 d. Nitrification and Denitrification pathways are key nitrogen cycling pathways that reduce the levels of ammonia nitrogen, and nitrite in the water. Correlation analysis in this study revealed a significant negative correlation between ammonia nitrogen changes and *Limnohabitans*, *Sediminibacterium*, and *Algoriphagus*, as well as a significant negative correlation between nitrite and *Roseomonas* and *Rubrivivax*. These microorganisms may be potential beneficial microorganisms for water quality improvement. However, the specific functions of these microorganisms in *C. nasus* aquaculture water, particularly in nitrogen cycling, still require further research. Our findings provide new insights into the role of probiotics in nitrogen removal and offer crucial insights into reducing nutrient loads in fish farming ponds.

## Data Availability

The sequences were submitted to the NCBI SRA database (PRJNA949598, https://www.ncbi.nlm.nih.gov/bioproject/PRJNA949598, accessed on 18 December 2023).

## Supplementary Materials

The following supporting information can be downloaded at: https://www.mdpi.com/article/10.3390/microorganisms12030627/s1, Table S1: Contents of EM used in the present study; Table S2: Ingredient composition and analysis of the experimental diets (%); Table S3: Statistical analysis of clean reads based on metagenome; Table S4: Statistical analysis of assembly results; Table S5: Contribution of genes related to nitrogen cycling pathways; Table S6: Contribution of genera related to nitrogen cycling pathways.
